# GSK3β is a key regulator of the ROS-dependent necrotic death induced by the quinone DMNQ

**DOI:** 10.1038/s41419-019-2202-0

**Published:** 2020-01-02

**Authors:** Sonia Ciotti, Luca Iuliano, Sebastiano Cefalù, Marina Comelli, Irene Mavelli, Eros Di Giorgio, Claudio Brancolini

**Affiliations:** Department of Medicine, Università degli Studi di Udine. P.le Kolbe 4, 33100 Udine, Italy

**Keywords:** Apoptosis, Preclinical research

## Abstract

Signaling pathways controlling necrosis are still mysterious and debated. We applied a shRNA-based viability screen to identify critical elements of the necrotic response. We took advantage from a small molecule (G5) that makes covalent adducts with free thiols by Michael addition and elicits multiple stresses. In cells resistant to apoptosis, G5 triggers necrosis through the induction of protein unfolding, glutathione depletion, ER stress, proteasomal impairments, and cytoskeletal stress. The kinase GSK3β was isolated among the top hits of the screening. Using the quinone DMNQ, a ROS generator, we demonstrate that GSK3β is involved in the regulation of ROS-dependent necrosis. Our results have been validated using siRNA and by knocking-out GSK3β with the CRISPR/Cas9 technology. In response to DMNQ GSK3β is activated by serine 9 dephosphorylation, concomitantly to Akt inactivation. During the quinone-induced pro-necrotic stress, GSK3β gradually accumulates into the nucleus, before the collapse of the mitochondrial membrane potential. Accumulation of ROS in response to DMNQ is impaired by the absence of GSK3β. We provide evidence that the activities of the obligatory two-electrons reducing flavoenzymes, NQO1 (NAD(P)H quinone dehydrogenase 1) and NQO2 are required to suppress DMNQ-induced necrosis. In the absence of GSK3β the expression of NQO1 and NQO2 is dramatically increased, possibly because of an increased transcriptional activity of NRF2. In summary, GSK3β by blunting the anti-oxidant response and particularly *NQO1* and *NQO2* expression, favors the appearance of necrosis in response to ROS, as generated by the quinone DMNQ.

## Introduction

In multicellular organisms cell death processes regulate organogenesis and tissue homeostasis. These forms of cellular demise have been generally considered as programmed cell death^1,2^. Noxious insults can also trigger cell death, as the result of an unmanageable damage. These varieties of cell elimination belong to the group of the accidental cell death^3,4^. Programmed and accidental cell deaths can be accomplished by the engagement of distinct signaling pathways, which activate both common and distinct molecular machineries devoted to cells elimination^1,5–7^. Apoptosis is a cell autonomous and evolutionary conserved genetic program, evolved to finalize a harmonious cellular disassembling.

In cells deficient for apoptosis cell death can still occur through alternative mechanisms. Moreover, certain stimuli can directly engage alternative death pathways such as ferroptosis or necroptosis^6–9^. Necrosis can also be triggered by noxious insults and apoptosis and necrosis can, in some conditions, co-exist. Despite the mechanisms controlling cell death by apoptosis are well-known, the existence of specific molecular players regulating necrosis is still debated^8^.

G5 is a non-selective isopeptidases inhibitor that can react with cellular thiols, thus eliciting multiple cellular stresses^10–15^. G5 belongs to a family of compounds that have been synthetized and investigated for the ability to commend accidental death in cancer cells, with therapeutic perspectives^16–22^. Protein misfolding, ER-stress, deubiquitinases inhibition and accumulation of poly-ubiquitylated proteins, glutathione depletion, alterations of the actin cytoskeleton and of the cell adhesion mark the cellular response to G5. In cells resistant to apoptosis this plethora of stresses can result in a necrotic death, which is distinguishable from necroptosis^11,13,14^. However, few additional data are available about the signaling networks transducing this peculiar form of necrosis.

G5, due to its pleiotropic effects, represents an ideal compound to identify genes controlling different stress signaling pathways that ultimately conduct to a necrotic death. For this reason, we conducted a functional shRNA-based screening aimed to identify genes that can influence cell survival in response to this compound. We used as a model the glioblastoma cell line U87MG, which activates necrosis when treated with high doses of G5^12,14^.

## Results

### shRNA screen to identify elements of the necrotic death induced by the proteotoxic stressor G5

To identify genes controlling G5-induced necrosis, we conducted a shRNA-based survival screen, using Cellecta’s Lentiviral shRNA Library Module 1. This module targets 5046 signaling pathway associated genes and consists of 27.500 shRNAs. For every target are present from 5 to 6 different shRNAs. U87MG cells were chosen as cellular model, because of their propensity to die by necrosis in response to G5^12^. The screen was based on the hypothesis that U87MG cells expressing a shRNA, targeting a gene necessary for the G5-induced cell death, would show a survival advantage and would be over-represented after sequencing.

**Fig. 1:**
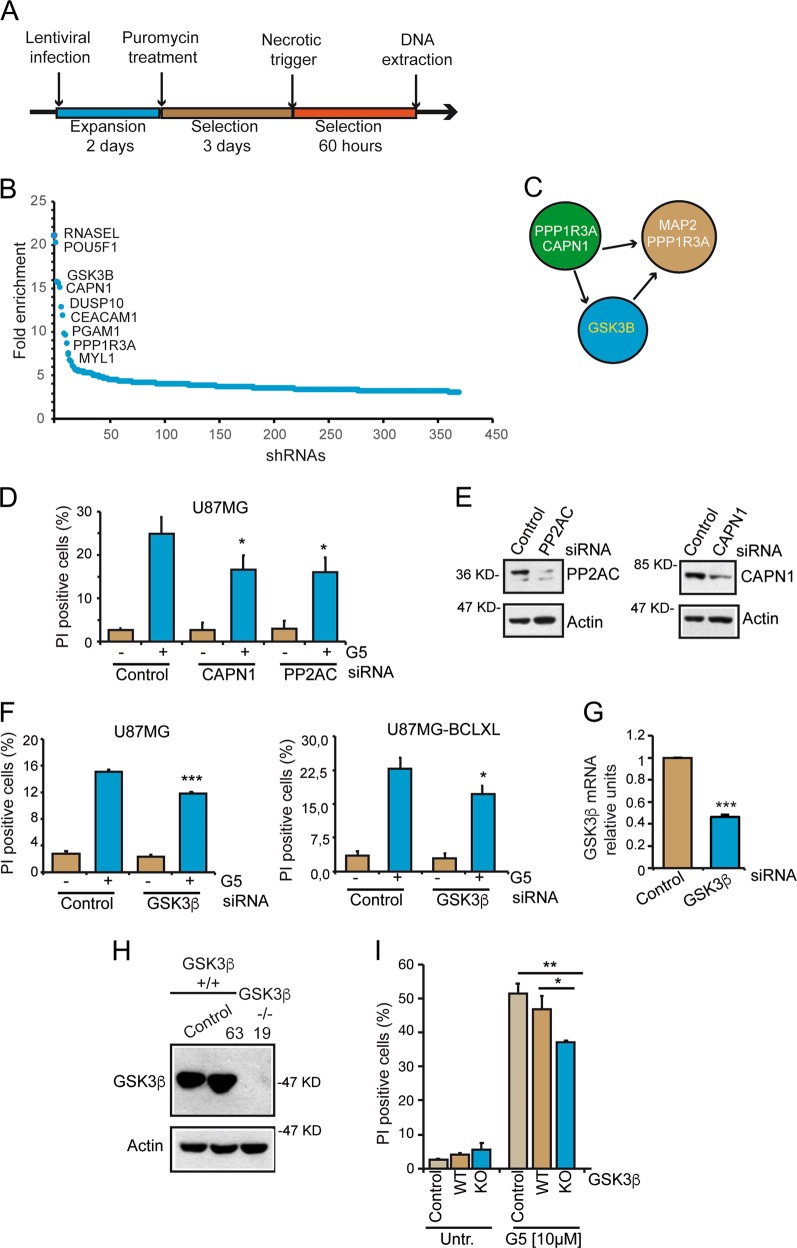
Screening protocol and targets validation. **a** Schematic representation of the screening protocol. 66 × 10^6^ millions of cells were infected with the lentiviral particles containing the shRNA plasmids with a MOI of 0,1 for 2 days. For the selection of the expressing-shRNAs, cells were first grown in the presence of puromycin (2 μg/ml) for additional 3 days and subsequently treated with G5 (2.5 μM) for 60 h. Surviving cells were harvested to recover the genomic DNA. **b** Representation of the most enriched hits. Data are represented as fold enrichment respect to the median of all shRNAs. The enrichment is relative to the second most abundant shRNA. Only shRNA with a fold increase >3 are shown. **c** Hits that are in relationship with GSK3β as up-stream regulators or downstream effectors. **d** U87MG cells were silenced for CAPN1 and PP2AC and, after 48 h from transfection, they were treated with G5 (2.5 μM) for 24 h. Cell death was calculated as percentage of cells positive to PI staining using cytofluorimetric analysis. Data are from three experiments; +SD. **e** Cellular lysates from the silenced cells were analyzed by immunoblot. Antibodies anti-CAPN1, anti-PP2AC, and anti-Actin (as loading control) were used as indicated. **f** U87MG and U87MG-BCLXL cells were silenced for GSK3β. After 48 h they were treated with G5 (2.5 μM) for further 24 h. Cell death was calculated as percentage of cells positive to PI staining using cytofluorimetric analysis. Data are from three experiments; +SD. **g** qRT-PCR analysis of GSK3β mRNA levels in silenced cells. Data were from three experiments; +SD. **h** Immunoblot analysis of GSK3β levels in the indicated clones of U87MG cells, selected after CRISPR/Cas9 mediated knock-out. Actin was used as loading control. **i** Cytofluorimetric analysis were performed to measure the % of PI positivity in the different U87MG clones treated with G5 (10 µM) for 24 h. Data are presented as mean ± SD. *n* = 3.

The infection and the selection scheme is described in Fig. 1a. Briefly, after 2 days of recovery from infections, cells were treated with puromycin and, after 3 days of selection, G5 was added for 60 h. Genomic DNA was extracted from the surviving cells and the abundance of every integrated shRNA-specific barcode was amplified by PCR with vector-specific primers and identified using high-throughput (HT) sequencing. The deconvolution and normalization of the reads for each barcode, respect to control shRNAs (against the Luciferase), has revealed the identity of the most enriched shRNAs. The relevant “hits” were defined when at least three different shRNAs were enriched in comparison to the median value of the control shRNAs targeting the luciferase (1541 reads) or the median value for all shRNAs (1490 reads). In order to isolate genes that could play key functions in transducing the necrotic signal, we imposed that the second shRNAs was to be enriched >3 fold compared to the median value. In this manner we selected 371 genes (Fig. 1b and Table S1). Among the top target genes we identified: *RNASEL*, *POU5F1*, *GSK3B*, *CAPN1*, and *DUSP10*. We focused the attention on glycogen synthase kinase-3 β (GSK3β) for two main reasons. First GSK3β can be activated under specific stress-conditions and it is involved in the regulation of cell death pathways in different cellular contexts^23–27^. Second, among other represented hits interacting proteins, regulators and substrates of GSK3β were found. These include Calpain-1 (CAPN1)^28^, the protein phosphatase-1 (PPP1)^29^, and the substrate MAP2 (Fig. 1c)^30^.

### Validation of CAPN1 and GSK3β as genes transducing G5-induced necrosis

The involvement of CAPN1 in different necrotic responses is well established^31^. To validate CAPN1 as an effector of the G5-induced necrosis, we silenced its expression using a siRNA targeting a different region of the gene. In addition, a siRNA against PP2AC was used as positive control^13^.

In comparison with the control transfected cells, U87MG cells silenced for CAPN1 or PP2AC were partially protected from G5-induced cell death (Fig. 1d). The potency of the silencing was confirmed by the reduction of the respective protein levels (Fig. 1e).

Next, we validated GSK3β as an element of the G5-induced cell death pathway. Its expression was silenced in U87MG and U87MG/BCL-XL cells, with a siRNA targeting a different sequence, respect to those recognized by the shRNAs isolated with the screening. U87MG/BCL-XL cells were used to further exclude the induction of apoptosis. In both cell lines the silencing of GSK3β only modestly decreased G5-induced necrosis (Fig. 1f).

The modest impact of GSK3β silencing in the necrotic pathway triggered by G5 could be the consequence of the low silencing efficiency. In fact, ~40% of the mRNA is still expressed after transfection (Fig. 1g). To unambiguously clarify this point, we applied the CRISPR/Cas9 system to knock-out GSK3β in U87MG cells (Fig S1a). After the screening of 253 clones, a *GSK3β*^−/−^ clone (number 19) was identified. The immunoblot analysis demonstrates the complete absence of the GSK3β protein in these cells in comparison with two clones of *GSK3β*^+/+^ cells, which underwent the same selection (Fig. 1h). The *GSK3β*^+/+^ control U87MG cells were infected with Cas9 without the sgRNA (indicated as control cells). Clone 63 was infected with both Cas9 and the sgRNA but resulted as a clone still WT for GSK3β, after the screening (indicated as WT cells). Genomic DNA Sanger sequencing of *GSK3β* loci in the three cell lines demonstrated the insertion of a T in the exon 1, two nucleotides after the PAM, in the KO clone and the presence of a WT GSK3β in the clone 63 and in the WT control (Fig. S1b). The insertion of a T causes the frameshift and the appearance of a STOP codon. Only the first 10 aa of GSK3β can be translated in the *GSK3β*^−/−^ cells. This event explains the absence of the GSK3β protein (Fig. 1h). Having proved that *GSK3β*^−/−^ cells do not show overt deficits in their proliferative capacity (Fig. S1c), U87MG/*GSK3β*^+/+^ and U87MG/*GSK3β*^−/−^ cells were treated with G5 for 24 h. Similarly to the silenced cells, U87MG/*GSK3β*^−/−^ cells acquired some resistance to G5-treatment (Fig. 1h). The moderate resistance to G5-treatment is evident only at high concentrations and after prolonged periods of treatment (24 h) with the drug (Fig. S2a, b). This observation further suggests the induction of a necrotic form of cell death. G5-induced cell death is poorly affected by inhibitors of necroptosis or of ferroptosis (Fig. S2a, b). In summary, GSK3β plays a partial contribution in transducing necrotic signals elicited by G5.

### GSK3β is a critical transducer of DMNQ-induced oxidative death

**Fig. 2:**
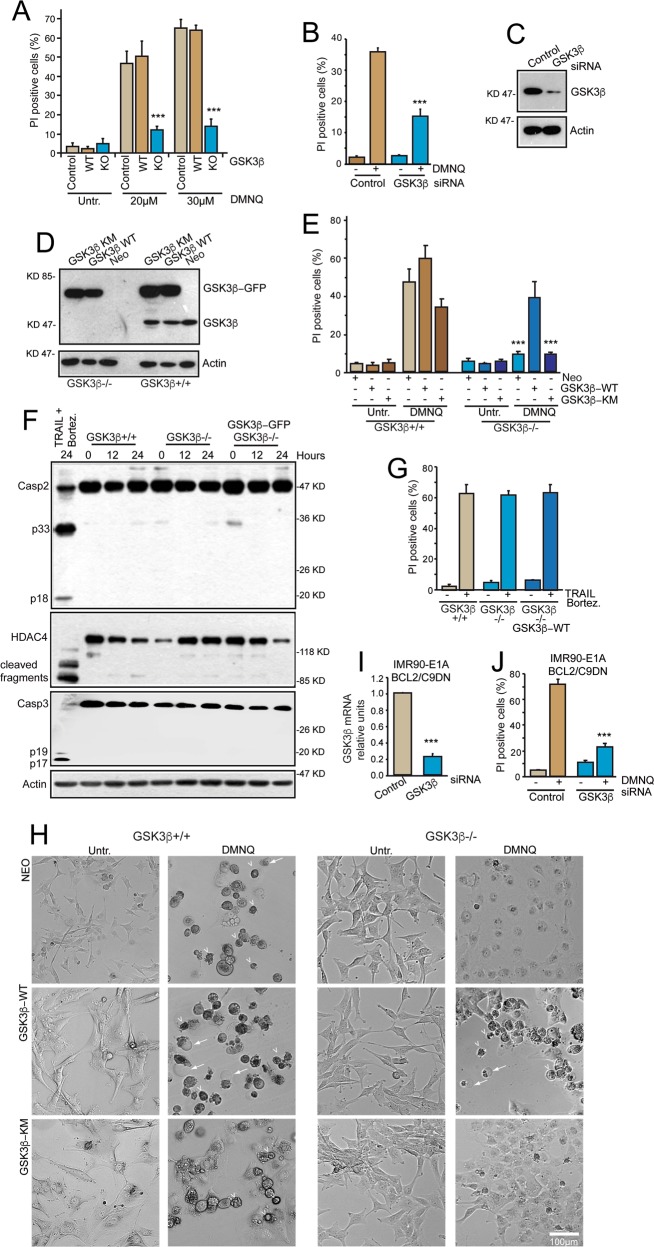
GSK3β is necessary for the necrotic response elicited by DMNQ. **a** Cytofluorimetric analysis measuring cell death percentages (PI positivity) in the different U87MG clones treated with the indicated concentrations of DMNQ for 24 h. Data are presented as mean ± SD. n = 3. **b** Cytofluorimetric analysis measuring cell death percentages (PI positivity). U87MG cells were transfected with siRNAs against GSK3β or control. After 48 h they were treated with 30 µM of DMNQ for further 24 h. Data are presented as mean ± SD. *n* = 3. **c** Immunoblot analysis of GSK3β levels in U87MG cells transfected with siRNAs against GSK3β or control. Actin was used as loading control. **d** Immunoblot analysis of GSK3β levels in U87MG *GSK3β*^+/+^ and *GSK3β*^−/−^ cells retrovirally infected with the GSK3β-GFP fusions WT or its catalytically inactive mutant K85A (KM). Actin was used as loading control. **e** Cytofluorimetric analysis measuring cell death percentages (PI positivity) in the indicated U87MG cell lines treated with 30 µM of DMNQ for 24 h. Data are presented as mean ± SD. *n* = 3. **f** Immunoblot analysis of Caspase-3, Caspase-2, and HDAC4 caspase-dependent processing in the indicated U87MG cell lines treated with 30 µM of DMNQ. Incubation with the combination TRAIL (2.5 ng/ml) and bortezomib (0.1 µM) for 20 h was used to trigger apoptosis. Actin was used as loading control. **g** Cytofluorimetric analysis measuring cell death percentages (PI positivity) in the indicated U87MG cell lines treated or not with the combination TRAIL (2.5 ng/ml) and bortezomib (0.1 µM) for 24 h. Data are presented as mean ± SD. *n* = 3. **h** Microscopic images of the indicated U87MG cell lines treated for 24 h with DMNQ (30 µM). Arrows point to membrane blistering and arrowheads to examples of necrotic cells. Phase contrast images were obtained with Leica DMi1 microscope with a 10x objective. **i** qRT-PCR analysis of GSK3β mRNA levels in the silenced IMR90-E1A//BCL2/C9DN cells. Data are from three experiments; +SD. **j** Cytofluorimetric analysis measuring cell death percentages (PI positivity). IMR90-E1A/BCL2/C9DN cells were transfected with siRNAs against GSK3β or control and after 48 h treated with 100 µM of DMNQ for further 24 h. Data are presented as mean ± SD. *n* = 3.

G5 triggers pleiotropic stresses, which can drive cells to death through different pathways. Proteasome impairment, misfolding and proteotoxic stress, oxidative stress and cytoskeletal malfunctions are all hallmarks of the G5-induced cell death^11–14^. The partial impact of GSK3β could stem from the co-existence of these multiple pathways. Since GSK3β can be involved in regulating ROS-induced cell death^32–35^, we hypothesized that its involvement in G5-induced death could depend on the ability of the compound of triggering oxidative stress. The impact of G5 on oxidative stress could be direct, through the depletion of glutathione or indirect, through the induction of protein misfolding^14^. To prove this hypothesis, we selected the redox-cycling 2,3-dimethoxy-1,4-naphthoquinone (DMNQ), a well-known inducer of oxidative stress. DMNQ toxicity is mediated by ROS production via one-electron-based redox cycling^36^. When U87MG cells were treated with DMNQ, *GSK3β*^−/−^ cells showed a strong resistance to death (Fig. 2a). To exclude a clone-specific effect, the contribution of GSK3β to DMNQ-induced cell death was validated by RNAi. DMNQ-induced cell death was effectively compromised also after GSK3β silencing (Fig. 2b). The decreased level of GSK3β in the corresponding silenced cells was confirmed by the immunoblot analysis (Fig. 2c). Finally, we proved that also menadione, another quinone, requires GSK3β to efficiently trigger cell death (Fig. S3a). In summary, GSK3β plays a partial role during G5-induced cell death and a fundamental role during DMNQ-induced cell death.

### GSK3β kinase activity is required for DMNQ-induced oxidative necrotic death

To further confirm the role of GSK3β in transducing DMNQ-induced oxidative death, we re-expressed GSK3β in U87MG/*GSK3β*^−/−^ cells. The GSK3β wild type (WT) and its kinase dead mutant (KM), carrying the K85A aa substitution were both C-terminal GFP-tagged. As control we also expressed GSK3β and its mutant in U87MG/*GSK3β*^+/+^ cells. Immunoblot analysis confirmed the expression of the different GSK3β fusions in *GSK3β*^−/−^ and *GSK3β*^+/+^ cells (Fig. 2d). The same cells expressing only the neomycin resistance gene were used as control (Neo cells). Cell death in response to DMNQ was recovered only in U87MG/*GSK3β*^−/−^ cells re-expressing the GSK3β-WT. Restoring only GSK3β expression without the catalytic activity (KM mutant) was insufficient to recover the necrotic defect of the KO cells (Fig. 2e). DMNQ-induced cell death is largely caspase-independent and it is marginally affected by the Ferrostatin-1, an inhibitor of ferroptosis (Fig. S2c, d and Fig. S3c). On the opposite it is strongly dependent on the activity of RIP1, as previously reported^13^. Hence, GSK3β kinase activity is necessary to trigger cell death in response to DMNQ.

GSK3β can also influence cell death by apoptosis^37^. Therefore, to confirm that DMNQ specifically elicits necrosis also in cells overexpressing the kinase, we evaluated caspases activation, the key enzymes of the apoptotic pathway. Caspase-3 and Caspase-2 activation and HDAC4 processing (a caspase-3 substrate) were monitored by immunoblot. Cells were also treated with the combination TRAIL/bortezomib a renowned apoptotic stimulus^38^. Figure 2f shows that GSK3β-dependent, DMNQ-induced cell death, does not require caspase activation. On the opposite, U87MG cells, after incubation with TRAIL/bortezomib, strongly activate caspases. We also demonstrated that GSK3β is not required for TRAIL/bortezomib-induced apoptosis (Fig. 2h). DMNQ-induced cell death showed the features of necrosis, with the appearance of vacuolization and membrane blistering^11^. The appearance of these necrotic features is strictly dependent on the kinase activity of GSK3β (Fig. 2h).

Next, we proved that the role of GSK3β in transducing a necrotic signal is not limited to U87MG cells. We took advantage from IMR90-E1A cells expressing Bcl-2 and a dominant negative mutant of Caspase-9. These engineered cells are resistant to apoptosis and die by necrosis^39^. The role of GSK3β in the DMNQ-dependent necrotic response was confirmed also in human fibroblasts. When GSK3β was silenced in these cells (Fig. 2i), DMNQ was unable of triggering cell death (Fig. 2j).

### DMNQ treatment activates GSK3β

To explore whether GSK3β is activated during DMNQ and G5-induced necrosis, the phosphorylation status of Ser-9 was evaluated. Once phosphorylated, Ser-9 inhibits the kinase activity of GSK3β, by acting as a pseudosubstrate^40,41^. Akt is the main up-stream regulator of GSK3β activity, through Ser-9 phosphorylation^40,42^. Hence, we also evaluated Akt activation levels by monitoring Thr-308 and Ser-473 phosphorylation status^13^.

**Fig. 3:**
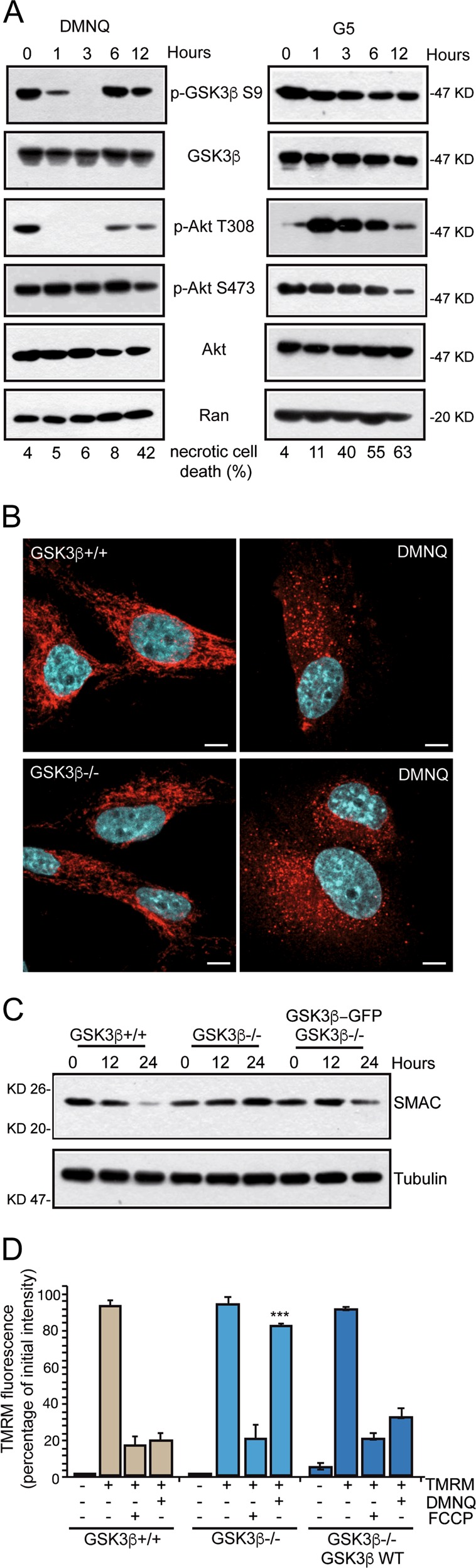
GSK3β is activated by DMNQ treatment. **a** U87MG cells were treated with 30 µM DMNQ or 10 µM G5 for the indicated times. Cellular lysates were generated and immunoblots were performed with the indicated antibodies. In parallel cell death was scored by cytofluorimetric analysis and it is showed at the bottom. **b** U87MG *GSK3β*^+/+^ and *GSK3β*^−/−^ cells were treated or not with 30 µM DMNQ for 24 h. Immunofluorescence analysis was performed to visualize mitochondria morphology, using anti-SMAC antibodies (red). Nuclei were stained with Hoechst 33258 (cyan). Bar 10 µm. Confocal images are shown in pseudocolors and were acquired with a Leica SP8 LSM. **c** Immunoblot analysis of SMAC levels in the indicated U87MG cells treated with 30 µM DMNQ for the indicated time points. Actin was used as loading control. **d** Cytofluorimetric analysis of TMRM fluorescence. The indicated U87MG cells were incubated for 30 min with TMRM (1 µM). FCCP (10 µM) was used for 5 min. Data are from three experiments. Columns represent the percentage of initial intensity of TMRM fluorescence + SD.

In DMNQ-treated cells an early and dramatic dephosphorylation of GSK3β Ser-9 was detected, as soon after 1 h from treatment, which became stronger after 3 h (Fig. 3a). The reappearance of Ser-9 phosphorylation after 6 h could mark the emerging of cells resistant to DMNQ treatment. Akt Thr-308 dephosphorylation parallels the behavior of Ser-9, thus suggesting a direct link between Akt inactivation and GSK3β activation in response to DMNQ. Akt Ser-473 phosphorylation remains unperturbed after DMNQ treatment (Fig. 3a).

Ser-9 dephosphorylation was observed also in response to G5. Here the decrease is modest at early times but appears more consistent at later time-points (12 h). This delayed activation of GSK3β parallels the inactivation of Akt (Fig. 3b). Overall, GSK3β activation, as monitored by Ser-9 phosphorylation, is modest in G5-treated compared to DMNQ-treated cells.

### DMNQ-dependent mitochondrial dysfunctions: the role of GSK3β

DMNQ treatment triggers mitochondrial fragmentation, which depends on Drp1 activities^13^. Smac/DIABLO localization was used to monitor the mitochondrial morphology and the integrity of the outer mitochondrial membrane (OMM)^43^. In *GSK3β*^−/−^ and *GSK3β*^+/+^ U87MG cells the mitochondrial networks were similar (Fig. 3b). DMNQ treatment triggers mitochondrial fragmentation in both cell lines, which is therefore independent from GSK3β. By contrast, SMAC levels were clearly reduced in U87MG/*GSK3β*^+/+^ compared to *GSK3β*^−/−^ cells (Fig. 3c). Re-expression of GSK3β promoted the decrease of SMAC protein. The degradation of SMAC in response to DMNQ could be related to its release into the cytoplasm as a consequence of the OMM rupture^39^. The alteration of mitochondrial functionality was confirmed by the measure of the mitochondrial membrane potential (Δψ_m_). After 24 h of DMNQ treatment, Δψ_m_ is completely collapsed in *GSK3β*^+/+^ cells but maintained in *GSK3β*^−/−^ cells (Fig. 3d). As control, incubation with the mitochondrial uncoupler FCCP triggered mitochondrial membrane depolarization in a GSK3β independent manner (Fig. 3d).

### Δψ_m_ dissipation and the nuclear translocation of GSK3β

GSK3β is a pleiotropic kinase involved in multiple signaling pathways. Although it is mainly a cytosolic protein, it can also localize in other subcellular compartments, including the nucleus and the mitochondria^44,45^. Particularly, GSK3β shuttles between the nucleus and the cytoplasm and a decline of PI3K/Akt activity can favor its nuclear accumulation^44^.

**Fig. 4:**
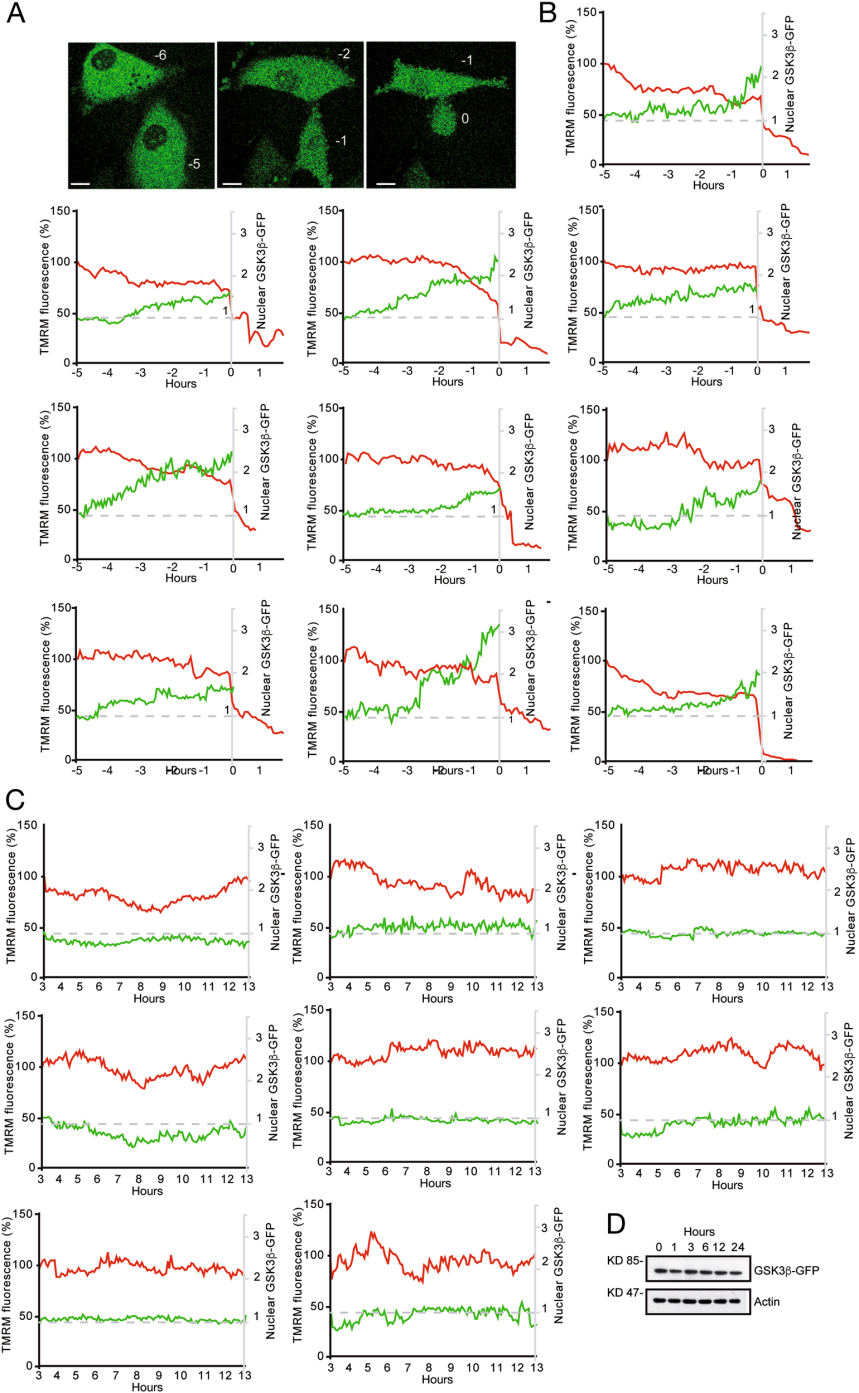
Nuclear translocation of GSK3β WT-GFP and mitochondrial Δψm dissipation at single cell level in vivo. **a** Representative frames of two U87MG/*GSK3β*^−/−^ cells re-expressing GSK3βWT-GFP treated with 30 µM DMNQ. Numbers indicate h before Δψ_m_ dissipation. Bar 16 µm. **b** Individual traces of cellular TMRM fluorescence (red) and of the nuclear/cytosolic fluorescence of GSK3βWT-GFP (green) in U87MG/*GSK3β*^−/−^ cells re-expressing GSK3βWT-GFP. Cells were treated with 30 µM DMNQ for 24 h. **c** Individual traces of cellular TMRM fluorescence (red) and of the nuclear/cytosolic fluorescence of GSK3βWT-GFP (green) in untreated U87MG/*GSK3β*^−/−^ cells, re-expressing GSK3βWT-GFP. **d** Immunoblot analysis of GSK3βWT-GFP levels in U87MG/*GSK3β*^−/−^ cells treated with 30 µM DMNQ for the indicated times. Actin was used as loading control.

To monitor the subcellular localization of GSK3β in vivo during necrosis, U87MG/*GSK3β*^−/−^ cells expressing the GSK3β-GFP were exposed to DMNQ and subjected to time-lapse confocal microscopy. We also measured in parallel the mitochondrial membrane potential, as a reference of the necrotic death. Figure 4a shows selected time-frames of the analysis, proving the progressive nuclear accumulation GSK3β-GFP, which becomes evident 1 h before the collapse of Δψ_m_. Figure 4b shows the individual traces for TMRM uptake and GSK3β-GFP localization in 10 typical U87MG/*GSK3β*^−/−^ cells expressing GSK3β-GFP, in response to DMNQ. In all examples the progressive nuclear accumulation of GSK3β-GFP anticipates Δψ_m_ collapse. By contrast, when the same analysis was performed in untreated cells, nuclear accumulation of GSK3β-GFP was not observed and only “physiological fluctuations” of the Δψ_m_^46^ were monitored (Fig. 4c). The integrity of the GSK3β-GFP chimera throughout the time of the analysis was verified by immunoblot (Fig. 4d). These data indicate that DMNQ triggers the nuclear accumulation of GSK3β-GFP before Δψ_m_ collapse.

### DMNQ-triggered ROS generation requires GSK3β activity

The quinone DMNQ triggers ROS generation after one-electron reduction by several intracellular flavoenzymes such as the NADPH-cytochrome P450 reductase or nitric oxide synthetases. The product of the one-electron reduction, DMNQ•-, reacts rapidly with O_2_ to form superoxide anion, O_2_•-, regenerating DMNQ. This redox-cycle is critical for redox signaling and toxicity^36,47^.

**Fig. 5:**
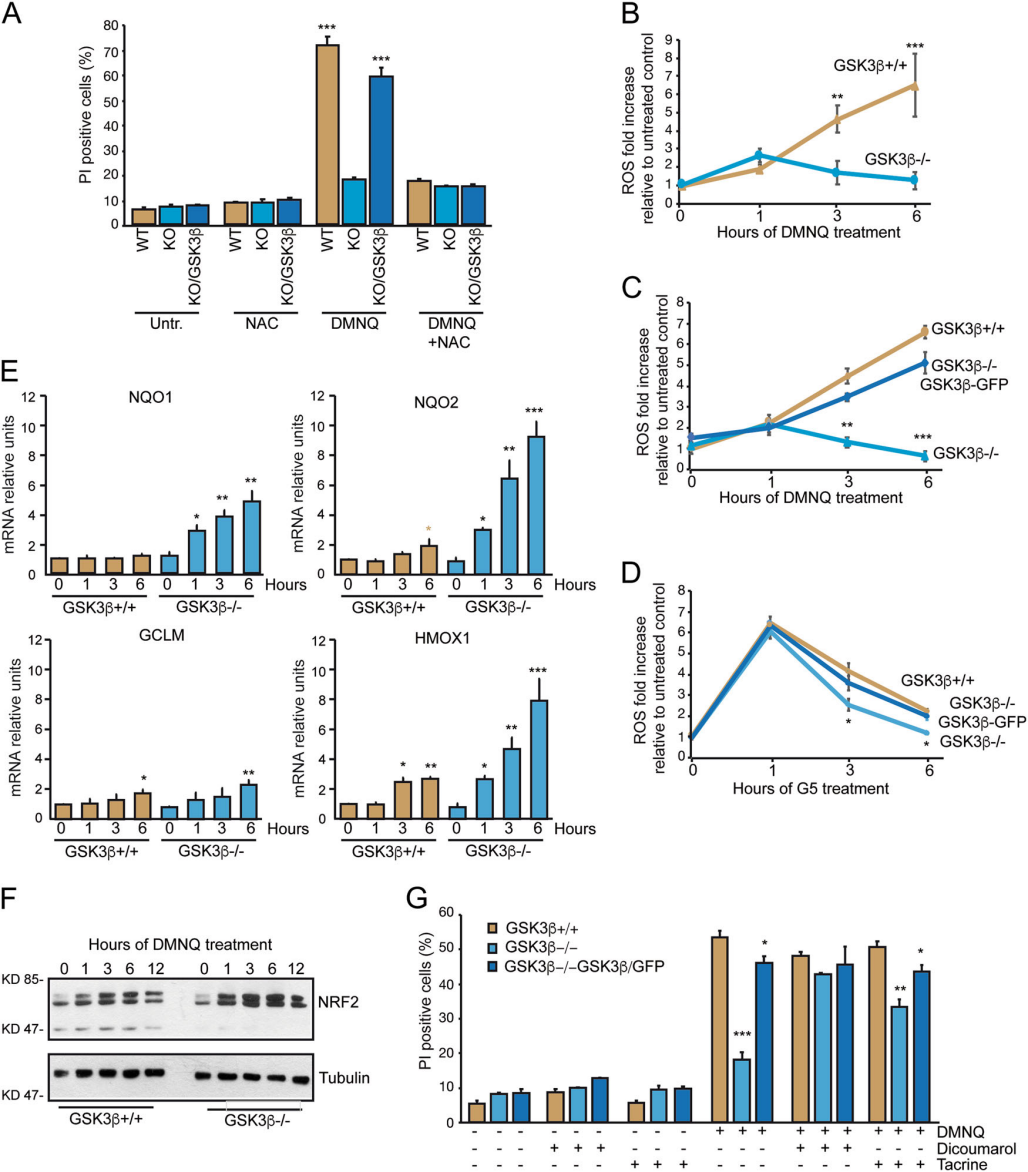
GSK3β favors ROS generation in response to DMNQ. **a** Cytofluorimetric analysis measuring cell death percentages (PI positivity) in the indicated U87MG cell lines, treated or not with the indicated combinations of molecules. DMNQ was used 30 µM. N-acetylcysteine (NAC) was 5 mM. Incubation was for 24 h. Data are presented as mean ± SD. *n* = 3. **b** Levels of reactive oxygen species as measured by Carboxy-H_2_DCFDA fluorescence in U87MG *GSK3β*^*+/+*^ and *GSK3β*^−/−^ cells treated with 30 µM DMNQ. **c** Levels of reactive oxygen species as measured by ROS Deep Red Dye fluorescence in the indicated U87MG cell lines treated with 30 µM DMNQ. **d** Levels of reactive oxygen species as measured by ROS Deep Red Dye fluorescence in the indicated U87MG cell lines treated with 10 µM G5. **e** Time-course analysis of *NQO1, NQO2, HMOX1* and *GCLM* mRNA expression levels. U87MG *GSK3β*^+/+^ and *GSK3β*^−/−^ cells were treated with 30 μM of DMNQ for the indicated times and the mRNA levels were monitored by qRT-PCR. Data are from three experiments; +SD. **f** Immunoblot analysis of NRF2 levels in U87MG *GSK3β*^+/+^ and *GSK3β*^−/−^ cells treated with 30 µM DMNQ for the indicated times. Actin was used as loading control. **g** Cytofluorimetric analysis measuring cell death percentages (PI positivity) in the indicated U87MG cell lines, treated or not with the indicated combinations of molecules. DMNQ was used 30 µM, Dicoumarol 100 µM, Tacrine 20 µM. Incubation was for 24 h. Data are presented as mean ± SD. *n* = 3.

Therefore, we explored whether GSK3β activity was required to sustain the redox cycle. First, we demonstrated that potentiation of the antioxidant properties, by treating cells with N-acetylcysteine (NAC), completely abrogates the GSK3β-dependent toxicity of DMNQ (Fig. 5a). Next, we compared DMNQ-induced ROS levels, using two different sensors: Carboxy-H_2_DCFDA and Deep Red Dye, in the presence or absence of GSK3β. DMNQ, through its redox-cycling activity triggers a progressive increase of ROS (Fig. 5b). In the absence of GSK3β, after an initial increase, ROS levels dropped-down, reaching a condition similar to the untreated cells. Re-expression of GSK3β restored the ROS accumulation in response to DMNQ (Figs. 5c and S4).

We also monitored the appearance of ROS in response to G5. Differently from DMNQ, G5 triggers a rapid increase of ROS, within 1 h from treatment, that declines at later times. the contribution of GSK3β is significant also in the case of G5-generated ROS. However, it is much less pronounced, when compared to DMNQ (Fig. 5d).

### ROS generation requires GSK3β activity to blunt the expression of NRF2-target genes involved in the anti-oxidant response

ROS generation by DMNQ can be prevented by obligatory two-electrons reducing flavoenzymes, such as NQO1 (NAD(P)H quinone dehydrogenase 1) and NQO2. These enzymes, by producing hydroquinones, prevents ROS generation by circumventing one-electron reductase-dependent redox cycling^47–49^. The strong impact of GSK3β on ROS generation could stem from its ability of influencing *NQO1* and *NQO2* activities. To verify this hypothesis, we analyzed the expression levels of the two enzymes in U87MG/*GSK3β*^−/−^ and *GSK3β*^+/+^ cells treated with DMNQ. qRT-PCR showed that only in response to DMNQ the expression levels of *NQO1* and *NQO2* dramatically increase in *GSK3β*^−/−^ cells. By contrast, only *NQO2* expression is modestly up-regulated in *GSK3β*^+/+^ cells (Fig. 5e). We also compared the expression of *HMOX1* and *GCLM*, two genes that belong to the anti-oxidant defense. *GCLM* is similarly and modestly up-regulated in the two cell lines whereas HMOX1 shows a behavior similar to *NQO2*.

The transcription of these genes in response to oxidative stressors is under the supervision of NRF2^50^. The activity of this transcription factor is subjected to multiple levels of controls, including half-life variations. In fact, NRF2 is subjected to proteasomal-mediated degradation, which is suppressed by the oxidative stress^50^.

As expected DMNQ triggers the up-regulation of NRF2. However, in the absence of GSK3β, this up-regulation is more pronounced and sustained through the time (Fig. 5f).

To demonstrate the key role of *NQO1* and *NQO2* in the necrotic response triggered by DMNQ, U87MG*/GSK3β*^+/+^, *GSK3β*^−/−^ *or GSK3β*^−/−^ expressing GSK3β-GFP were treated with dicoumarol a potent inhibitor of NQO1 and tacrine, a recently identified NQO2 inhibitor^51,52^. The resistance to DMNQ-induced necrosis observed in the absence of GSK3β was completely abolished by the inhibition of NQO1, as well as, after the inhibition of NQO2, although less efficiently (Fig. 5g).

In summary, GSK3β, through the regulation of NRF2 levels, can blunt the expression of *NQO1* and *NQO2*, two enzymes that prevent ROS generation. In this manner GSK3β can promote cell death by necrosis.

## Discussion

Quinone compounds are ubiquitously diffused in the environment as elements of the food chains or as air pollutants, for example in the diesel exhaust particles^53^. Quinones can also be generated in the body as a result of some xenobiotic metabolism through the cytochrome P450 system^36^. DMNQ has been intensively studied as an example of quinones toxicity. DMNQ toxicity is mediated by ROS production via one-electron-based redox cycling. Several flavoenzymes, including NADPH-cytochrome P450 reductase and NADH-cytochrome b reductase, can fulfil the one-electron reduction of DMNQ^36,47,48^. For this reason DMNQ is also commonly used, as a tool, to investigate the cellular responses to the oxidative stress.

**Fig. 6:**
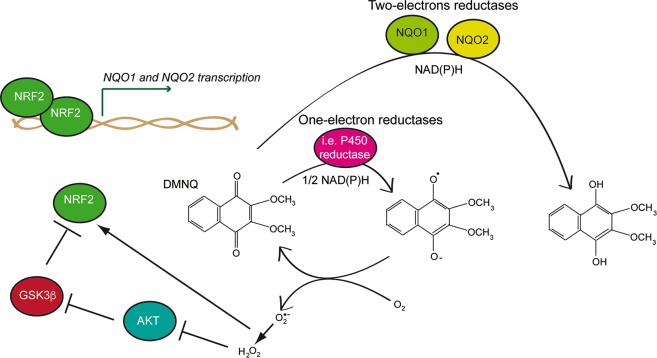
Graphical summary of the role of GSK3β during DMNQ-induced necrotic cell death.

In this manuscript we have demonstrated that GSK3β is a key player of the DMNQ/ROS-induced necrotic death. We show that GSK3β is responsible for reducing the anti-oxidant response engaged by NRF2. This response leads the up-regulation of the obligatory two-electron reducing flavoenzymes NQO1 and NQO2^47,48,51,52^. These flavoenzymes are required to blunt the redox cycling activity of DMNQ. In the absence of GSK3β, NRF2 levels and the transcription of its targets *NQO1* and *NQO2* is strongly sustained. In this manner the cytotoxic effect of quinones is nullified (Fig. 6).

Our results suggest a model where high ROS levels, as generated by DMNQ, inhibit AKT^13,54^ and consequently unleash GSK3β activity, which in turn, switches-off the NRF2 anti-oxidant responses^50,55,56^. Hence, the impact of GSK3β in this necrotic pathway is mainly exerted by suppressing a pro-survival signal (Fig. 6).

The described pathway is pathological relevant and it is implicated in other models of cell death elicited by oxidative stress. Examples are the ischemia and reperfusion injury in the brain^57^, in hepatocytes^58,59^, during diabetic nephropathies^60^, in a model for Alzheimer disease^61^ and in other models of neurological diseases^62,63^.

The time-lapse analysis suggests that the DMNQ-dependent nuclear accumulation of GSK3β anticipates the Δψ_m_ collapse. Hence, it is plausible that GSK3β activation is coupled to its nuclear accumulation where it phosphorylates NRF2, a signal necessary for its nuclear exclusion, its poly-ubiquitylation and the subsequent proteasomal degradation^44,64^.

GSK3β was identified after a high-throughput shRNA screening, aimed to define new players of the necrotic response induced by G5. Unexpectedly, the kinase plays only a minor role in this form of death. In agreement with our observation GSK3β activation is much less evident in response to G5 compared to DMNQ. Even though we have demonstrated that G5 is able to trigger oxidative stress.

We suggest that Akt could explanation this apparent paradox. Akt in response to G5 is not dephosphorylated at early time points, differently from DMNQ. Instead we confirmed a strong increase of Thr 308 phosphorylation, as previously reported^13^. The two cysteine residues 310 and 296 found in the T-loop region are critical for Akt activity. We have recently shown that G5 can directly target Akt, possibly by reacting with these cysteines residues^15^. Hence G5 could directly interfere with Akt activities. How it could occur and which could be the consequence on Akt activity, deserve further investigations.

Finally, we have not completely unveiled the molecular basis of the necrotic pathway elicited by G5. GSK3β and the oxidative stress probably play only a secondary or additive role in this pathway. During the investigation of the role of GSK3β, we also tested the contribution of other genes, which were included among the top hits of the screening. Transfection of isolated siRNAs only minimally reduced G5-induced cell death. It is plausible that, the simultaneous induction of multiple stresses by G5 causes multiple cellular dysfunctions that ultimately trigger necrosis. Under this condition, ablation of a single gene is not enough to rescue cells from the death commitment.

## Materials and methods

### Cell culture conditions and reagents

The cell lines used in this article were Uppsala 87 Malignant Glioma (U87MG) glioblastoma cell line, IMR90-E1A lung fibroblast cell line, Human Embryonic Kidney cells 293 T1 (HEK293T1) cell line and Phoenix Amphotropic (AMPHO) embryonic kidney cell line. All cell lines were cultured at 37 °C in 5% CO_2_ atmosphere in Dulbecco's Modified Eagle's Medium (DMEM), (Sigma-Aldrich) supplemented with 10% fetal bovine serum (FBS); glutamine (2 mmol/L), penicillin (100 U/mL) and streptomycin (100 μg/mL) (Euroclone). All cell lines were tested for mycoplasma contamination and resulted as mycoplasma free. U87MG cells were authenticated by gene expression profile. U87MG/BCL-XL and IMR90/E1A/BCL-2/C9DN cells, expressing a dominant negative mutant of caspase-9 were previously described^11,12^ and verified for the expression of the transgenes. The following chemicals were used: 4H-thiopyran-4-one, tetrahydro-3,5-bis[(4-nitrophenyl) methylene]-1,1-dioxide (G5)^10^; 2,3-dimethoxy-1,4-naphthoquinone (DMNQ); carbonilcyanide *p*-triflouromethoxyphenylhydrazone (FCCP); NAC; Propidium Iodide (PI), Ferrostatin-1 and DMSO (Sigma Aldrich); Tetramethyl Rhodamine Methyl-ester (TMRM) (Life Technologies); Bortezomib (LC Laboratories); TNF-related apoptosis-inducing ligand (TRAIL)^65^; G418 (Euroclone); Hygromycin (PanReac-AppliChem); Dicoumarol and Tacrine (Santa Cruz Biotechnology); Necrostatin-1 (Enzo Life Sciences); Boc-D-FMK (Abcam).

### shRNA library screening

U87MG cells were transduced with DECIPHER Pooled shRNA library-Human Module 1 (Cellecta, Mountain View, CA, USA) composed by 27500 shRNAs targeting 5043 genes (5–6 shRNAs/mRNA). The HTS3 (DECIPHER pRSI9-U6-(sh)-HTS3-UbiC-TagRFP-2A-Puro-dW) cassette of each shRNA contains the U6 RNA polymerase III promoter to drive shRNAs expression, the fluorescence protein (RFP) and the puromycin resistance. All shRNAs have unique 18-nucleotide DNA barcode sequences, which facilitate their identification after HT sequencing. The HEK293T1 packaging cells were transfected with 60 μg of the plasmid shRNAs library and 300 μg of the packaging plasmid mix (Cellecta Inc., psPAX2: pMD2.G), in DMEM without serum or antibiotics and in the presence of Plus Reagent^TM^ and Lipofectamin^TM^ (Life Technologies). The concentrated lentiviral particles were re-suspended in PBS with 10% FBS and stored at −80 °C for the lentiviral titer estimation. After the calculation of the Transduction Units, 66 × 10^6^ cells were transduced with the shRNA library at MOI of 0.1 to ensure that ~90% of the cells are infected with one shRNA-carrying virus. After 48 h, infected cells were selected by adding Puromycin (2 μg/ml) for 72 h. Later, the selected cells were treated with G5 (2.5 μM) for 60 h. Genomic DNA (gDNA) was purified from the surviving cells using the QIAamp DNA Micro Kit (Qiagen, Hilden, Germany). Pooled barcodes were PCR-amplified from 100 μg of gDNA and identified after Illumina sequencing by deconvolution analysis. Positive hits were selected as genes when at least two different shRNAs scored higher frequencies in comparison to the average of the control luciferase (*n* ≥ 3).

### RNA interference

The RNA interference (RNAi) was performed using the following siRNAs direct against: CAPN1 (Santa Cruz Biotechnology); PP2Ac (Life Technologies); GSK3β (Fw 5′-GCAUUUAUCGUUAACCUAA-3′, Rv 5′-UUAGGUUAACGA UAAAUGC - 3′, Sigma Aldrich); or the relative control siRNAs. U87MG, U87MG/BCL-XL and IMR90/E1A/BCL-2/C9DN cells were transfected 24 h after seeding. Six hours later the medium was changed, and after 48 h of silencing, cells were treated with G5 or DMNQ for further 24 h. RNA was extracted and the protein lysates were collected for the subsequent analysis.

### Drug treatments, PI- and TMRM-assay

For cytofluorimetric analysis, drug treated cells were collected in PBS and incubated with propidium iodide (PI) for 5 min at room temperature. PI fluorescence was determined by the FACSCalibur flow cytometer (BD, San Jose, CA,) at the excitation wavelength of 585 nm. For TMRM assay, cells were incubated with TMRM (1 μM) for 30 min. TMRM fluorescence was determined by flow cytometer at the excitation wavelength of 488 nm.

### Cell lysis and Western Blotting

The cellular lysis was performed using an SDS denaturing lysis solution in which the protease inhibitor cocktail (PIC), phenylmethane sulfonyl fluoride (PMSF), Na_3_VO_4_ and β-mercaptoethanol were added. After SDS/PAGE electrophoresis proteins were transferred to a 0.2 µm-pore-sized nitrocellulose membrane. Immunoblotting was performed as previously described^11^. The used primary antibodies were: anti-PP2Ac (Upstate, 05-545); anti-actin (Sigma-Aldrich, A2066); anti-CAPN1 (sc-271313), anti-GSK3β (sc-377213) and anti-NRF2 (sc-365949) (Santa Cruz Biotechnology); anti-p-GSK3β S9 (9336), anti-p-Akt T308 (4056); anti-p-Akt T473 (9271), anti-Akt (9272), anti-Caspase-3 (9662) (Cell Signaling Technology), anti-Smac/DIABLO^39^ and anti-HDAC4^66^; anti-Caspase-2^67^; anti-tubulin^67^. The same membranes were incubated with the horseradish peroxidase-conjugated secondary antibody for 1 h at room temperature. The used secondary antibodies were goat anti-mouse or goat anti-rabbit (Sigma Aldrich). Finally, the blots were developed using Super Signal West Dura as recommended by the vendor (Pierce Waltham, MA, USA).

### RNA extraction and qRT-PCR

Cells were lysed using Tri-Reagent (Molecular Research Center). 1.0 μg of total RNA was retro-transcribed by using 100 units of M-MLV Reverse transcriptase (Life Technologies). qRT-PCRs were performed using SYBR green technology (KAPA Biosystems). Data were analyzed by comparative threshold cycle using *HPRT* and *GAPDH* as normalizer.

### Generation of GSK3β knock-out U87MG cells

U87MG cells null for GSK3β were achieved using the CRISPR/Cas9 technology. The single guide RNA (sgRNA) 5′-CCTTTGCGGAGAGCTGCAAG-3′ was designed using “CRISPR design” tool (http://crispr.mit.edu/). Lentiviral infections and selections were performed as previously described^68^. The KO clones were screened by PCR, immunoblots and validated by Sanger sequencing.

### Generation of U87MG/GSK3β^−/−^ cells expressing GSK3β WT and its mutant K/M fused to GFP

The coding sequence of GSK3β was amplified by PCR from Vectors encoding wild-type and kinase-dead GSK3β previously described^69^, using the following primers (Sigma-Aldrich): - AGATCTATGTCAGGGCGGCCCAG, as forward primer containing a restriction site for *Bgl*II; - GAATTCTGGTGGAGTTGGAAGCTGATG, as reverse primer containing the *Eco*RI site. GSK3β WT and the kinase defective mutant KM were cloned into pEGFP-N1 plasmid. Next the two fusions (GSK3β/WT-GFP and GSK3β/KM-GFP) digested *Bgl*II and *Xho*I were subcloned into the retroviral vector pWZL-Neo. U87MG cells expressing BCL-XL, GSK3β-WT-GFP or GSK3β/KM-GFP constructs were generated by retroviral infection as previously described^12^. G418 (1000 μg/ml) for the selection of GSK3β-WT-GFP or KM-GFP expressing cells and Hygromycin (200 μg/ml) for the selection of BCL-XL expressing cells were used. As control U87MG cells were infected with pWZL-Hygro and pWZL-Neo retroviral vectors and selection performed as above described.

### Immunofluorescences and time-lapse microscopy

U87MG cells were fixed with 3% paraformaldehyde and permeabilized with 0.5% Triton X-100. The primary antibody was anti-Smac/DIABLO, and the secondary antibody was Alexa Fluor 546-conjugated anti-rabbit (Life Technologies). Cells were imaged with a Leica confocal microscopy SP2 or SP8.

For time-lapse analysis TMRM (20 nM)^39^ was used and DMNQ (30 μM) added 1 h before the analysis. The images were collected every 5 min for 24 h using a Leica SP8 confocal microscope (Leica Microsystems) equipped with a stage top incubator controlling temperature, CO_2_ and humidity (Okolab). Image analysis was performed using the Leica Acquired Software X (LASX). For the TMRM Δψ_m_ analysis, the fluorescence of the mitochondria was evaluated through drawing a region around the cell (Region Of Interest; ROI) and measuring its Mean fluorescence Intensity (MI_TMRMcell_). The fluorescence of the background (BK_TMRM_), which is another ROI located in a non-fluorescent region, was subtracted to the MI_TMRMcell_ and the obtained data was multiplied for the area of the cell (Area_cell_), according to the following equation:

$${\mathrm{TMRM}}\,\mathrm{fluorescence}\;\left(\% \right) = \left( {\mathrm{MI}_{\mathrm{TMRMcell}} - \mathrm{BK}}_{\mathrm{TMRM}} \right) \ast \mathrm{Area}_{\mathrm{cell}}$$

A similar calculation was used to quantify the increase of nuclear fluorescence of GSK3β-GFP, according to the following formula:

$$\begin{array}{ll}\mathrm{Nuclear}\,\mathrm{GSK3}\beta - \mathrm{GFP} &= \left( {\mathrm{MI}_{\mathrm{GSK3}\beta - \mathrm{GFPnucl}} - \mathrm{BK}_{\mathrm{GSK3}\beta - \mathrm{GFP}}} \right) \ast \mathrm{Area}_{\mathrm{nucl}}\\ &\quad/\left( {\mathrm{MI}_{\mathrm{GSK3}\beta - \mathrm{GFPcytosol}} - \mathrm{BK}_{\mathrm{GSK3}\beta - \mathrm{GFPcytosol}}} \right) \ast \mathrm{Area}_{\mathrm{cytosol}}\end{array}$$

### ROS accumulation measurement

The reactive oxygen species (ROS) accumulation was evaluated using two different probes following the manufacturer’s instructions: 6-carboxy-2',7'-dichlorodihydrofluorescein diacetate (Carboxy-H_2_DCFDA) (Life Technologies) and the ROS Deep Red Dye from the Cellular Reactive Oxygen Species Detection Assay Kit Deep Red Fluorescence (Abcam). Carboxy-H_2_DCFDA and ROS Deep Red Dye fluorescence were determined by the FACSCalibur flow cytometer (BD) at the excitation wavelength of 495 nm for Carboxy-H_2_DCFDA and 650 nm for the ROS Deep Red Dye.

### Statistics

Results were expressed as means ± standard deviations (SD) from at least three independent experiments. Statistical analysis of differences between groups was performed using the Student’s *t* test of Excel software (two-samples, two-tailed distribution, equal variance), with *p* values represented as: *p < 0.05; **p < 0.01; ***p < 0.005. Measurements were obtained in double-blind.

## Supplementary information

Text

Figure S1

Figure S2

Figure S3

Figure S4

Table S1
