# Supplementary material for: GSK3β is a key regulator of the ROS-dependent necrotic death induced by the quinone DMNQ
Source: Cell Death Dis. 2020 Jan 2;11(1):2. doi: 10.1038/s41419-019-2202-0 (PMC6952365; doi:10.1038/s41419-019-2202-0)
Supplement: Supplementary file 1 — Text [file 41419_2019_2202_MOESM1_ESM.docx]

SUPPLEMENTAL MATERIALS

FIGURES LEGENDS

**Figure S1. Generation and characterization of U87MG cells knocked-out for GSK3B.**

A) Schematic representation of GS3KB genomic region target of CRISPR/Cas9 approach. The PAM sequence together with sgRNA utilized for the CRISPR approach are indicated. The genomic sequence of the WT clone and of the knock out clone 19 used in this study are reported.

B) Deducted aminoacid sequence of the WT clone and the knocked-out clone 19. The short amino-acid sequence generated by the frameshift mutation and the stop codon introduced are highlighted.

C) The different U87MG cell lines were grown in DMEM supplemented with 10% FBS for the indicated days. Mean ± SD; n = 3.

**Figure S2. Definition of the cell death pathways engaged by G5 or DMNQ in a GSK3β dependent manner**

A) Analysis of the cell death percentage, by measuring the PI positivity in the different U87MG cell lines expressing or not GSK3β. Cells were treated with the indicated concentrations of G5 for 24 hours in the presence or not of Necrostatin-1 or Ferrostatin-1.

B) Analysis of the cell death percentage, by measuring the PI positivity in the different U87MG cell lines expressing or not GSK3β. Cells were treated for the indicated times with 10µM of G5, in the presence or not of Necrostatin-1 or Ferrostatin-1.

C) Analysis of the cell death percentage, by measuring the PI positivity in the different U87MG cell lines expressing or not GSK3β. Cells were treated with the indicated concentrations of DMNQ for 24 hours, in the presence or not of Necrostatin-1 or Ferrostatin-1.

D) Analysis of the cell death percentage, by measuring the PI positivity in the different U87MG cell lines expressing or not GSK3β. Cells were treated with the indicated the indicated times with 30µM of DMNQ, in the presence or not of Necrostatin-1 or Ferrostatin-1.

The Countess™ II Automated Cell Counter (ThermoFisher Scientific) was used for the quantification of the PI positivity. When Necrostatin-1 or Ferrostatin-1 were used, cells were pre-incubated for 1 hour with the inhibitors. Data are presented as mean ± S.D. n=3. Red asterisks refer to the comparison of the same cell lines in the presence or not of the inhibitors. Black asterisks refer to the comparison of the same condition between cells expressing or not GSK3β.

**Figure S3.** **GSK3β regulates caspase-independent DMNQ and Menadione induced cell death manner**

A) Analysis of the cell death percentage, by measuring the PI positivity in the different U87MG-GSK3β^^ cells re-expressing or not GSK3β. Cells were treated with the indicated concentrations of DMNQ or menadione for 24 hours.

B) Analysis of the cell death percentage, by measuring the PI positivity in the different U87MG-GSK3β^^ cells re-expressing or not GSK3β. Cells were treated with the indicated concentrations of G5 or with 10µM of G5 for the indicated times. Boc-D-FMK was used 50µM. Cells were pre-incubated for 1 hour with the caspase inhibitor before treatments. Incubation with the combination TRAIL (2.5ng/ml) and bortezomib (0.1µM) for 24 hours was used to trigger apoptosis.

C) Analysis of the cell death percentage, by measuring the PI positivity in the different U87MG-GSK3β^^ cells re-expressing or not GSK3β. Cells were treated with the indicated concentrations of DMNQ or with 10µM of DMNQ for the indicated times. Boc-D-FMK was used 50µM. Cells were pre-incubated for 1 hour with the caspase inhibitor before treatments.

The Countess™II Automated Cell Counter (ThermoFisher Scientific) was used for the quantification of the PI positivity. Data are presented as mean ± S.D. n=3.

**Figure S4. ROS generation in response to DMNQ treatment.**

U87MG-*GSK3β^^ *and U87MG U87MG-*GSK3β^^ *cells re-expressing or not GSK3β were treated or not with 30µM DMNQ for the indicated times and then stained with ROS Deep Red dye in a 5% CO2, 37 °C incubator for 1 hour. The fluorescent intensities were measured with a FACSCalibur flow cytometer (BD) at the excitation wavelength of 650 nm..

**Table S1. Summary of the top 371 genes identified after the shRNAs screening**
