# Supplementary figures and images for: GSK3β is a key regulator of the ROS-dependent necrotic death induced by the quinone DMNQ
Source: Cell Death Dis. 2020 Jan 2;11(1):2. doi: 10.1038/s41419-019-2202-0 (PMC6952365; doi:10.1038/s41419-019-2202-0)

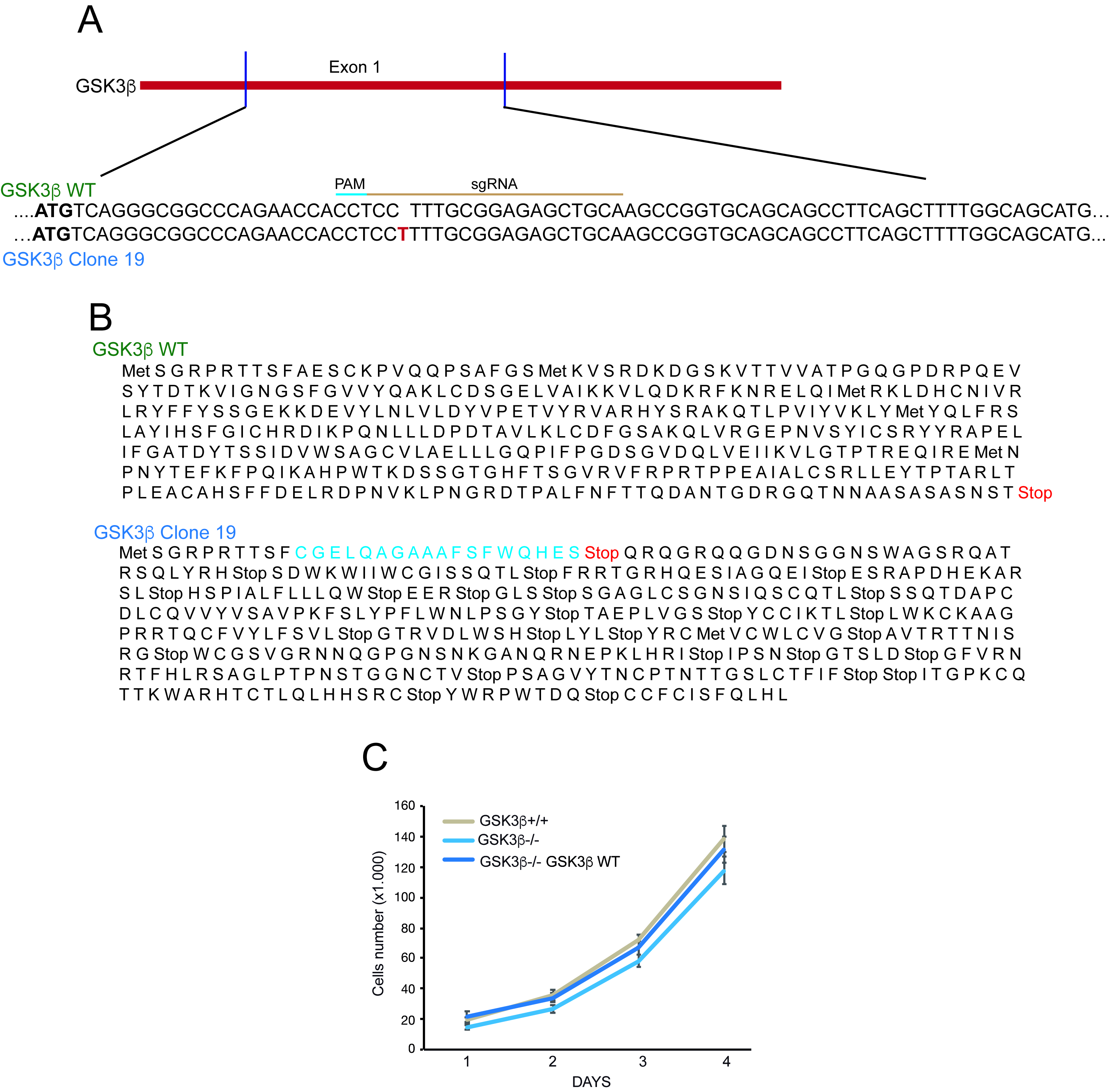

Supplement: Supplementary file 2 — Figure S1 [file 41419_2019_2202_MOESM2_ESM.tif]

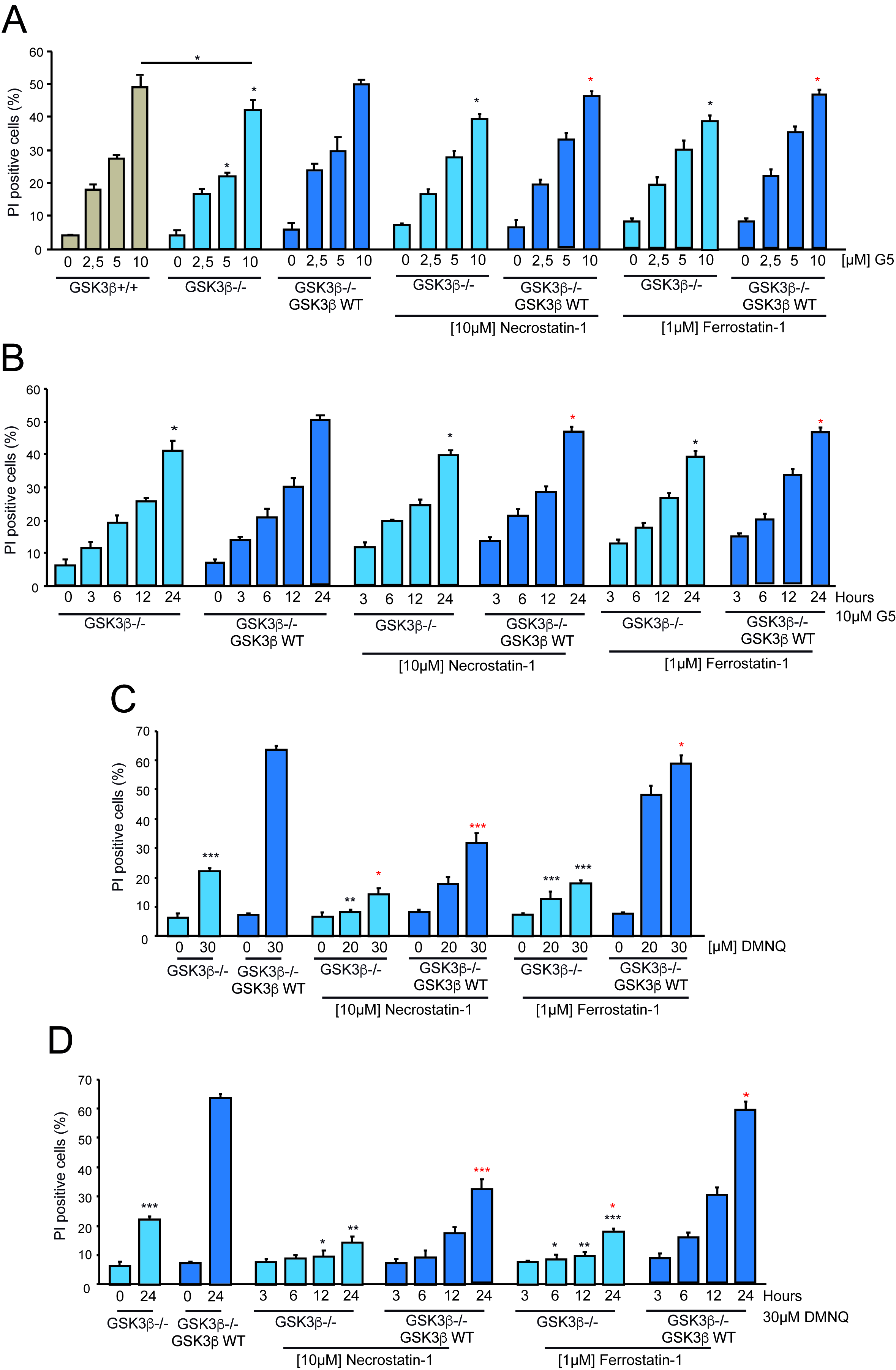

Supplement: Supplementary file 3 — Figure S2 [file 41419_2019_2202_MOESM3_ESM.tif]

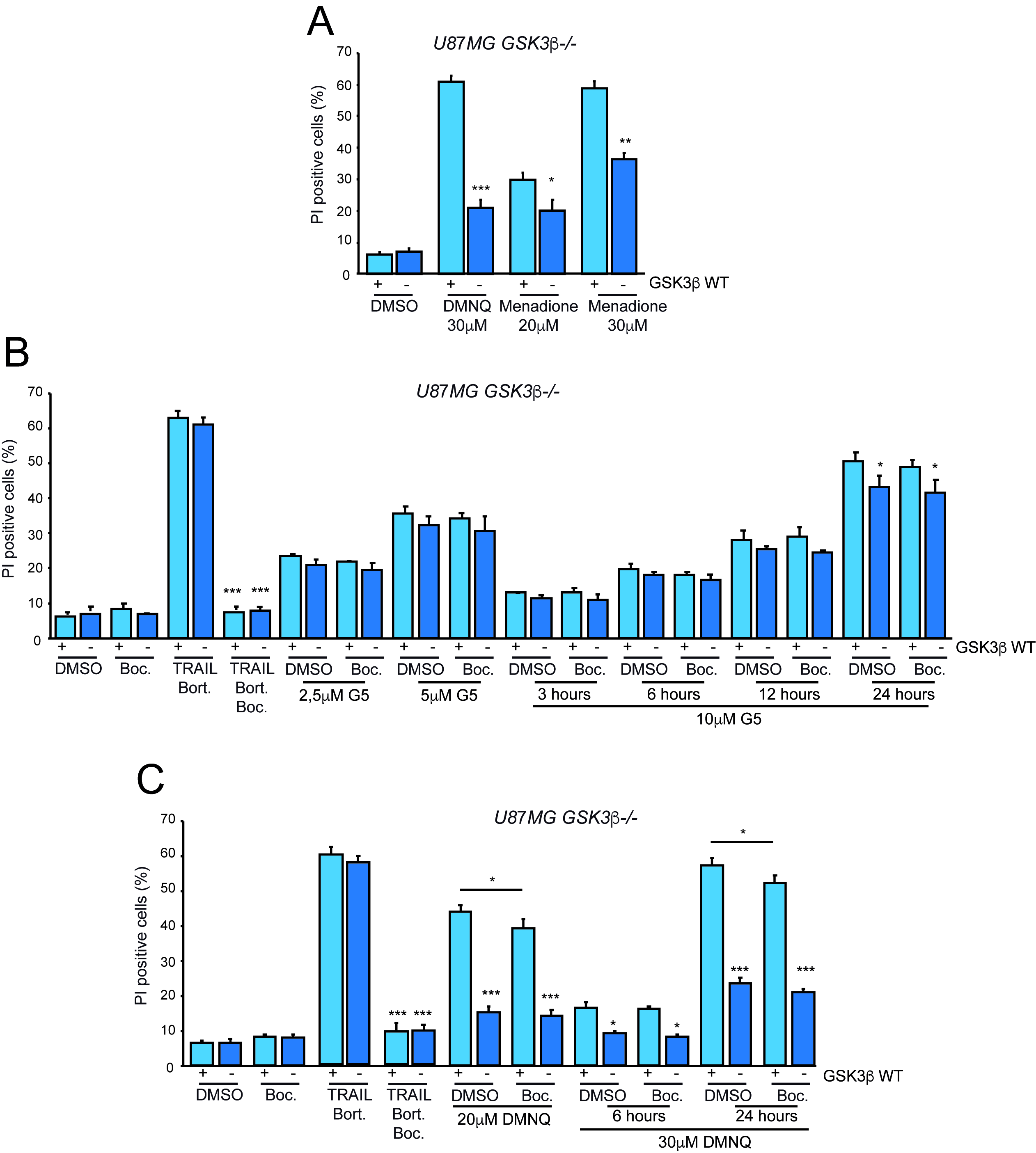

Supplement: Supplementary file 4 — Figure S3 [file 41419_2019_2202_MOESM4_ESM.tif]

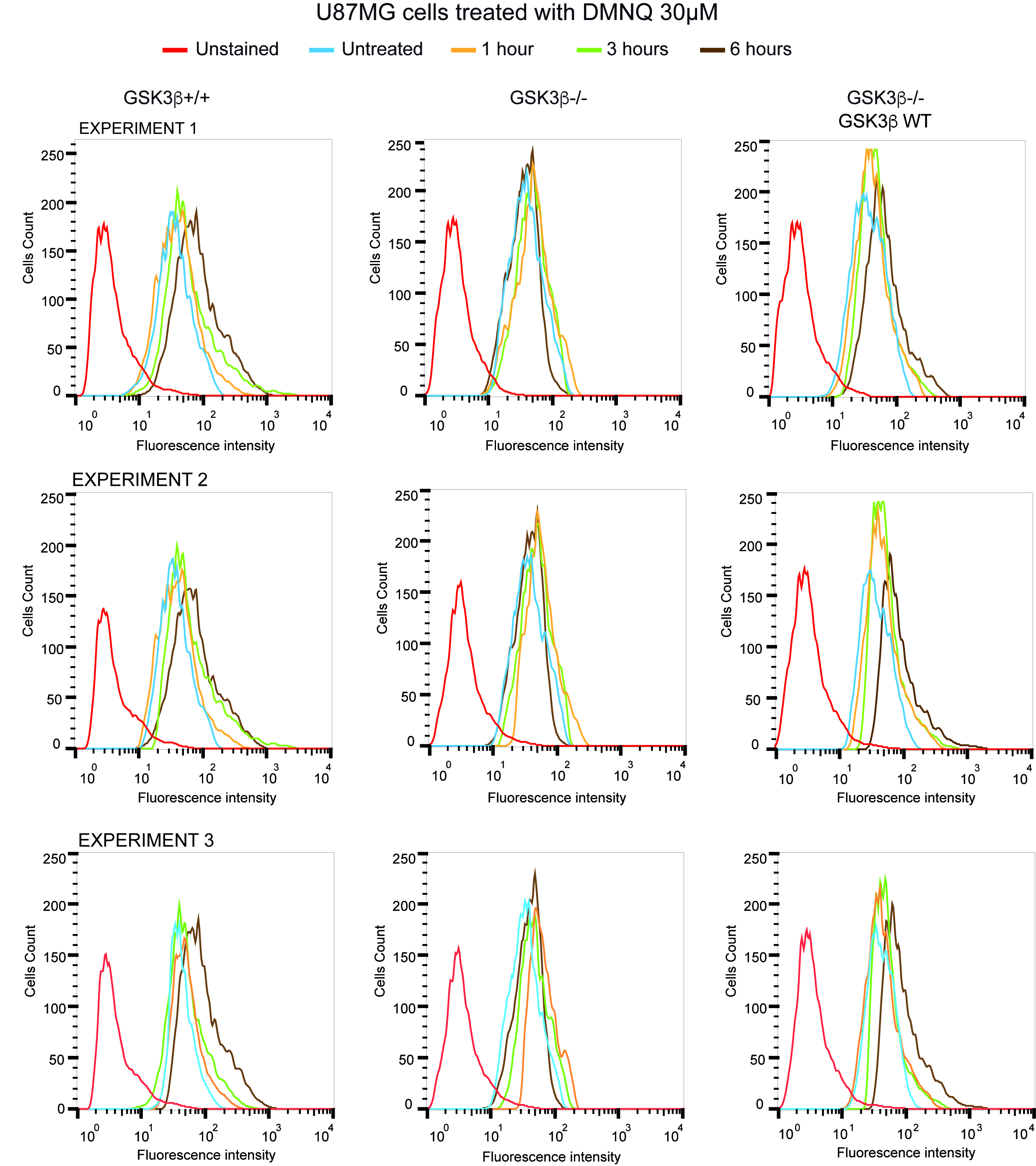

Supplement: Supplementary file 5 — Figure S4 [file 41419_2019_2202_MOESM5_ESM.tif]
